# Risk factors for lymphatic filariasis in two villages of the Democratic Republic of the Congo

**DOI:** 10.1186/s13071-019-3428-5

**Published:** 2019-04-11

**Authors:** Cédric B. Chesnais, Naomi-Pitchouna Awaca-Uvon, Johnny Vlaminck, Jean-Paul Tambwe, Gary J. Weil, Sébastien D. Pion, Michel Boussinesq

**Affiliations:** 1 IRD UMI 233, INSERM U1175, Université de Montpellier, Unité TransVIHMI, 911 Avenue Agropolis, P.O. Box 64501, 34394 Montpellier, France; 2 Programme National de Lutte contre les Maladies Tropicales Négligées à Chimiothérapie Préventive, Ministère de la Santé Publique, 36, Avenue de la Justice, C/Gombe, Kinshasa, Democratic Republic of the Congo; 3 Department of Virology, Parasitology and Immunology, Ghent University, Merelbeke, Belgium; 4 Infectious Diseases Division, Department of Internal Medicine, Washington University School of Medicine, 660 S. Euclid Ave., Campus Box 8051, St. Louis, MO USA

**Keywords:** Africa, Congo, Filariasis, Epidemiology, Risk factors, Bednets, Community study, Lymphatic filariasis

## Abstract

**Background:**

Little is known regarding risk factors for lymphatic filariasis (LF) in Central Africa. To expand on what is known, we studied the epidemiology of LF in two endemic villages in the Democratic Republic of the Congo.

**Methods:**

Dependent variables were *Wuchereria bancrofti* antigenaemia detected with filarial test strips (FTS) and microfilaraemia detected by night blood smears. The following factors were investigated: sex, age, the use of bednets, the use of latrines, hunting, fishing and agricultural activities, history of treatment with anthelmintic drugs, overnight stays in the bush, population density, the number of household members, and distance to rivers. Mixed multivariate logistic regression models were used.

**Results:**

Two hundred and fifty nine out of 820 (31.6%) of subjects aged ≥ 5 years had *W. bancrofti* antigenaemia and 11.8% (97/820) had microfilaraemia. Multivariable analysis of risk factors for antigenaemia demonstrated increased risk for males (aOR = 1.75, 95% CI: 1.20–2.53, *P* = 0.003), for older individuals (aOR = 9.12 in those aged > 35 years, 95% CI: 4.47–18.61, *P* < 0.001), for people not using bednets (aOR = 1.57, 95% CI: 1.06–2.33, *P* = 0.023), for farmers (aOR = 2.21, 95% CI: 1.25–3.90, *P* = 0.006), and for those who live close to a river (aOR = 2.78, 95% CI: 1.14–6.74, *P* = 0.024). Significant risk factors for microfilaraemia included age, male gender, overnight stay in the bush, and residence close to a river (aOR = 1.86, 2.01, 2.73; *P* = 0.011, 0.010, 0.041; for the three latter variables, respectively). People who reported having taken levamisole (*n* = 117) during the prior year had a significantly decreased risk of having filarial antigenaemia (aOR = 0.40, 95% CI: 0.21–0.76, *P* = 0.005).

**Conclusions:**

Age, sex, not using bednets, and occupation-dependent exposure to mosquitoes were important risk factors for infection with *W. bancrofti* in this study. The association with levamisole use suggests that the drug may have prevented filarial infections. Other results suggest that transmission often occurs outside of the village. This study provides interesting clues regarding the epidemiology of LF in Central Africa.

## Background

Lymphatic filariasis (LF), a major neglected tropical disease (NTD), is a mosquito-borne parasitic infection caused by *Wuchereria bancrofti*, *Brugia malayi* and *B. timori*. The Global Programme to Eliminate Lymphatic Filariasis (GPELF) was launched in 2000 with the goal of eliminating LF as a public health problem by 2020. To date, more than 7 billion treatments have been distributed during annual mass drug administration (MDA) campaigns [1]. MDA is no longer required in 21 of 72 endemic countries, and 11 of these have been validated by the World Health Organization (WHO) as having eliminated LF as a public health problem [1].

LF elimination programmes are not as advanced in Central Africa as in other regions, although there have been improvements in MDA coverage in recent years. Initially, the delay observed in Central Africa was due to (i) a lack of accurate epidemiological and geographical information on LF distribution [2–5]; (ii) concern about the potential risk of serious adverse events after ivermectin treatment in areas where *Loa loa* is co-endemic [6]; and (iii) insecurity and political instability in some countries. In addition, large-scale mapping surveys conducted in recent years revealed that LF distribution in this region is highly focal [5] and that the total population requiring MDA for LF was lower than expected [7]. Furthermore, the circulating filarial antigen (CFA) detection test used to map LF sometimes produces false positive results in individuals with high *Loa loa* microfilarial densities [8]. Thus, mapping for LF based on CFA testing overestimated the extent of LF in some areas (e.g. in Cameroon) [9].

In a previous paper, we investigated risk factors associated with *W. bancrofti* infection in a village in the Republic of Congo where baseline infection level was moderate (CFA prevalence: 17%) [5]. That study showed that overnight stays in the bush for hunting or fishing activity was a significant risk factor for filarial infection. The present study was conducted to assess risk factors in villages located in the western part of the Democratic Republic of the Congo (DRC) where LF prevalence was much higher than in the Republic of Congo study. This additional study is important, because it adds significant new information on individual risk factors for LF in Central Africa.

## Methods

### Context of the study

This study was carried out with baseline data collected in June 2014 during a community-based study to assess the impact of biannual mass administration of albendazole on LF [10]. In July 2013, community surveys were performed in 13 villages of the Kwilu Province. The survey area was delineated by Bandundu-ville in the west, Beno in the east, and the Kasaï and Kwilu rivers in the north, until 20 km south. Filarial antigenaemia rates were strongly heterogeneous, and Mbumkimi and Misay were the only villages where the prevalence of antigenaemia reached 40%.

### Study site

**Fig. 1:**
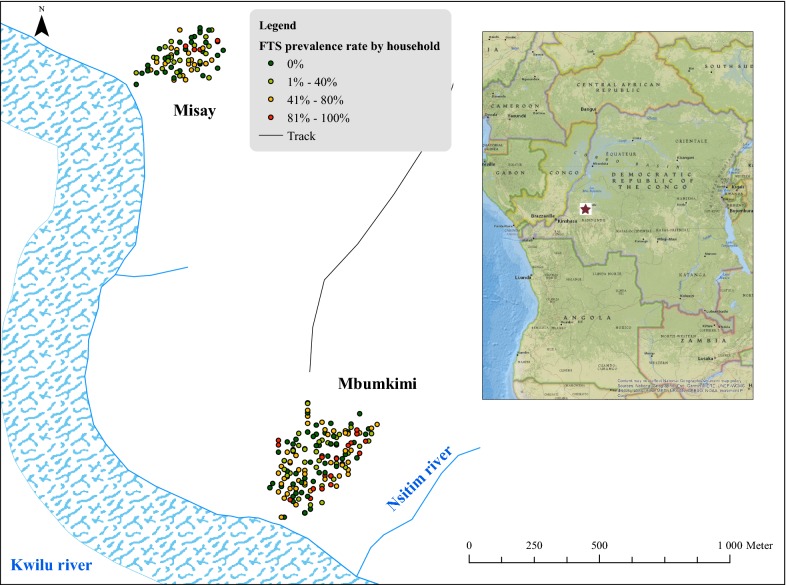
Study area of Misay and Mbumkimi. Each point indicates the location of a household. The red star indicates the location of the villages

The study was conducted in two villages, Mbumkimi and Misay (3°31′41″S, 17°37′44″E and 3°30′44″S, 17°37′29″E, respectively) in the Bagata territory of Kwilu Province, about 50 km east of the capital city of the province, Bandundu-ville. The villages are located less than 2 km apart, and both are located on the northern bank of the Kwilu River. The river is approximately 200 m wide near the study villages. In addition, a stream several meters wide, called Nsitim, flows into the Kwilu River approximately 300 m east of Mbumkimi (Fig. 1). The vegetation is comprised of forest savanna with gallery forests along the rivers. There are two seasons: a dry season from May to October, and a rainy season from November to April. The populations of Mbumkimi and Misay, as assessed by a census conducted in May 2014, were 843 and 423, respectively. The main occupational activities in the study villages are agriculture and fishing.

### Survey methods

In June-July 2014, adults and children ≥ 5 years of age were invited to participate in the community survey. Participants who signed a written consent form (assent plus written consent from at least one parent was required for children < 18 years of age) were screened for CFA using the Alere Filariasis Test Strip (FTS, Scarborough, ME, USA) [11]. Capillary blood was collected by finger prick using a sterile disposable lancet in a hematocrit tube. The blood (70 μl) was then transferred on the sample application pad of the strip following the manufacturer’s instructions. A single trained person read all of the tests at 10 min, and results were recorded as negative or positive. Participants with a positive FTS result were re-sampled between 10:00 PM and midnight to prepare two thick blood smears (70 µl) for detection of microfilariae (mf). Slides were dehaemoglobinized, stained with Giemsa within 24 h, and read by two independent microscopists. The arithmetic mean count from the two slides was recorded as mf density (expressed in mf/70 μl). All individuals with a negative FTS result were considered to be amicrofilaraemic.

### Questionnaire

A standardized questionnaire was used to collect demographic information (full name, age, sex), information on the peridomestic environment, and the individual’s activities outside the immediate village environment. Questions on the peridomestic environment asked about access to latrines (yes/no) and use of bednets during the previous night (yes/no) as a proxy for regular usage of bednets. Other questions included: hunting (yes/no), fishing (yes/no), agricultural activities (in three categories: none; yes, in fields located on the same side of the Kwilu River; and yes, in fields located across the Kwilu River). Participants were also asked if they occasionally stayed overnight in the bush (no/yes). Participants were also asked whether they had taken anthelmintic drugs within the previous year (yes/no); if the answer was yes, the name of the drug was recorded.

### Environmental assessment

Houses were georeferenced using GPS on smartphones. Geospatial data were collected in World Geodetic System (WGS) 1984 geographical coordinates and projected using the WGS 1984 World Mercator coordinate reference system. The accuracy of georeferencing was 4 m. Roads and rivers were also mapped.

### Statistical analysis

#### Dependent variables

Analyses were conducted using CFA and microfilaraemia (mf) as dependent variables.

#### Explanatory variables

The following individual factors were coded as categorical variables: village of residence (Mbumkimi or Misay), sex (M/F), use of latrines (yes/no), use of bednet (yes/no), hunting (yes/no), fishing (yes/no), occasional overnight stays in the bush (yes/no), agricultural activities (no; yes, in fields located on the same side; yes, in fields located across the Kwilu River), and history of anthelmintic treatment (yes/no). Age was categorized into 4 balanced classes: 5–10; 11–20; 21–40; and > 40 years. The number of members for each household was included as a quantitative variable. Regarding the environmental variables, distances (as the crow flies) between each house and each river (Kwilu and Nsitim rivers) were calculated categorized using ArcGIS v.10.4 (ESRI, Redlands, CA, USA) as follows: 0–300; 300–400; and > 400 m. The GPS location was unavailable for 24 households, corresponding to 60 individuals (7.3% of the study population), and these were considered to be missing data. Lastly, we defined the population density (in the confines around the outermost houses) using ArcGIS (inhabitants per km^2^), and then, each residence was categorized into three balanced levels (low, intermediate, and high population density).

#### Risk factors associated with filarial infection

Bivariate analyses were conducted using Chi-square tests to evaluate differences between FTS positive and negative individuals and between individuals with and without microfilaraemia. A multivariable analysis including all variables associated with CFA or mf at *P* < 0.25 by bivariate analysis was performed using a descending procedure. We used mixed multivariable logistic regression models with household as random effect on the intercept. From the final models, we estimated the population attributable fractions (PAF) for each independent variable by the presence of CFA and mf (i.e. we estimated the proportional reduction in the prevalence of CFA and mf if the risk factor was absent). This was performed using the *punaf* package in STATA software. All analyses were performed using the STATA v.12.1 software (StatCorps, LP, College Station, TX, USA).

## Results

### Study population

A total of 1290 people, including 1033 aged ≥ 5 years, were recorded in the two villages during the census. About 100 individuals could not be registered (e.g. due to refusal), and thus the total population in the two villages was about 1400. Of the 1290 people recorded, 820 subjects [including 383 males (46.7%) living in 255 households] were tested for filarial antigenaemia by FTS. Their median age was 20 years (range: 5–79 years, interquartile range: 10–35 years). Parasitological results are shown in Table 1 and univariate analyses of risk factors for *W. bancrofti* infection are shown in Table 2. Among participants who reported having taken anthelmintic drugs during the past year (*n* = 223), 117 had taken levamisole, 53 mebendazole, 52 albendazole, and 1 pyrantel.

**Table 1:** *Wuchereria bancrofti* prevalence and intensity of infection in Misay and Mbumkimi

Age (years)	Total					Males					Females				
	*n*	FTS-positive (%)	mf-positive (%)	*W. bancrofti* mf count per 70 μl		*n*	FTS-positive (%)	mf-positive (%)	*W. bancrofti* mf count per 70 μl		*n*	FTS-positive (%)	mf-positive (%)	*W. bancrofti* mf count per 70 μl	
				GM	Median (range)				GM	Median (range)				GM	Median (range)
5–10	227	8.4	1.8	20.1	25.5 (3–120)	116	6.9	1.7	21.3	25.5 (11.5–39.5)	111	9.9	1.8	19.0	61.5 (3–120)
11–15	119	28.6	10.9	25.8	30.5 (0.5–162.5)	67	12.4	10.5	18.6	22 (2.5–162.5)	52	28.9	11.5	37.9	82 (0.5–139.5)
16–20	73	27.4	8.2	3.4	5.3 (0.5–16.5)	32	37.5	12.5	5.8	8.3 (1–16.5)	41	19.5	4.9	1.2	1.8 (0.5–3)
21–30	136	38.2	14.7	9.2	17.3 (0.5–74)	54	44.4	24.1	8.3	14.5 (1–74)	82	34.2	8.5	11.3	20 (0.5–58)
31–40	112	47.3	16.1	13.9	11.8 (1.5–239.5)	49	61.2	26.5	13.7	14 (1.5–239.5)	63	36.5	7.9	14.4	10.5 (5.5–87.5)
41–50	76	57.9	25.0	9.1	12 (0.5–359.5)	30	66.7	33.3	6.2	3.8 (0.5–359.5)	46	52.2	19.6	13.9	19.5 (1.5–45)
> 50	77	48.1	22.1	14.5	21 (0.5–93)	35	51.4	17.1	18.7	26.8 (2–69.5)	42	45.2	26.2	12.6	12.5 (0.5–93)
Total	820	31.6	11.8	12.0	14 (0.5–359.5)	383	34.2	14.4	10.8	14 (0.5–359.5)	437	29.3	9.6	13.7	16.3 (0.5–139.5)

*Abbreviations*: *n*, number of subjects; FTS-positive, individuals with positive filarial test strip result; mf-positive, individuals with microfilaraemia; mf, microfilarial; GM, geometric mean microfilaraemia in microfilaremic individuals

**Table 2:** Univariate analysis of risk factors for *Wuchereria bancrofti* antigenaemia

	Total (*n *= 820)	Negative FTS (%)	Positive FTS (%)	*P*	Males	Negative FTS (%)	Positive FTS (%)	*P*	Females	Negative FTS (%)	Positive FTS (%)	*P*
Village												
Mbumkimi	543	359 (66.1)	184 (33.9)	0.047	259	161 (62.2)	98 (37.8)	0.030	284	198 (69.7)	86 (30.3)	0.535
Misay	277	202 (72.9)	75 (27.1)		124	91 (73.4)	33 (26.6)		153	111 (72.6)	42 (27.4)	
Bednets												
No	319	211 (66.1)	108 (33.9)	0.264	163	104 (63.8)	59 (36.2)		156	107 (68.6)	49 (31.4)	0.468
Yes	501	350 (69.9)	151 (30.1)		220	148 (67.3)	72 (32.7)	0.479	281	202 (71.9)	79 (28.1)	
Latrines												
No	150	103 (68.7)	47 (31.3)	0.941	63	39 (61.9)	24 (38.1)	0.476	87	64 (73.6)	23 (26.4)	0.513
Yes	670	458 (68.4)	212 (31.6)		320	213 (66.6)	107 (33.4)		350	245 (70.0)	105 (30.0)	
Fishing												
No	375	295 (78.7)	80 (21.3)	<0.001	207	151 (73.0)	56 (27.0)	0.001	168	144 (85.7)	24 (14.3)	0.001
Yes	445	266 (59.8)	179 (40.2)		176	101 (57.4)	75 (42.6)		269	165 (61.3)	104 (38.7)	
Hunting												
No	817	561 (68.7)	256 (31.3)	–	380	252 (66.3)	128 (33.7)	–	437	309 (70.7)	128 (29.3)	–
Yes	3	0	3 (100)		3	0	3 (100)		0	0	0	
Occasional overnight stay in the bush												
No	656	483 (73.6)	173 (26.4)	<0.001	294	217 (73.8)	77 (26.2)	<0.001	362	266 (73.5)	96 (26.5)	0.005
Yes	164	78 (47.6)	86 (52.4)		89	35 (39.3)	54 (60.7)		75	43 (57.3)	32 (42.7)	
Agricultural activity												
No	249	216 (86.8)	33 (13.2)	<0.001	146	126 (86.3)	20 (13.7)	<0.001	103	90 (87.4)	13 (12.6)	<0.001
Yes, fields same side	131	92 (70.2)	39 (29.8)		48	33 (68.8)	15 (31.2)		83	59 (71.1)	24 (28.9)	
Yes, fields other side	440	253 (54.5)	187 (42.5)		189	93 (49.2)	96 (50.8)		251	160 (63.8)	91 (36.2)	
Previous anthelmintic treatment												
No	557	362 (65.0)	195 (35.0)	0.003	251	158 (63.0)	93 (37.0)	0.224	306	204 (66.7)	102 (33.3)	0.001
Yes	223	173 (77.6)	50 (22.4)		122	89 (70.5)	36 (29.5)		101	87 (86.1)	14 (13.9)	
na	40	26 (65.0)	14 (35.0)		10	8 (80.0)	2 (20.0)		30	18 (60.0)	12 (40.0)	
Population density around the household												
Low	186	129 (69.3)	57 (30.7)	0.421	84	56 (66.7)	28 (33.3)	0.991	102	73 (71.6)	29 (28.4)	0.254
Intermediate	262	188 (72.8)	74 (28.2)		119	79 (66.4)	40 (33.6)		143	109 (76.2)	34 (23.8)	
High	312	205 (65.7)	107 (34.3)		152	99 (65.1)	53 (34.9)		160	106 (66.2)	54 (33.8)	
na	60	39 (65.0)	21 (35.0)		28	18 (64.3)	10 (35.7)		32	21 (65.6)	11 (34.4)	
Distance to Nsitim River												
> 400 m	472	340 (72.0)	132 (28.0)	0.034	223	158 (70.9)	65 (29.1)	0.026	249	182 (73.1)	67 (26.9)	0.159
300–400 m	243	155 (63.8)	88 (36.2)		114	64 (56.1)	50 (43.9)		129	91 (70.5)	38 (29.5)	
0–300 m	45	27 (60.0)	18 (40.0)		18	12 (66.7)	6 (33.3)			15 (55.6)	12 (44.4)	
Distance to Kwilu River												
> 400 m	98	64 (65.3)	34 (34.7)	0.001	46	26 (56.5)	20 (43.5)	0.010	52	38 (73.1)	14 (26.9)	0.048
300–400 m	232	181 (78.0)	51 (22.0)		109	84 (77.1)	25 (22.9)		123	97 (78.9)	26 (21.1)	
0–300 m	430	277 (64.4)	153 (35.6)		200	124 (62.0)	76 (38.0)		230	153 (66.5)	77 (33.5)	

*Abbreviations*: Negative and Positive FTS, individuals with negative and positive filarial test strip results, respectively; na, not available

### Filarial antigenaemia

**Fig. 2:**
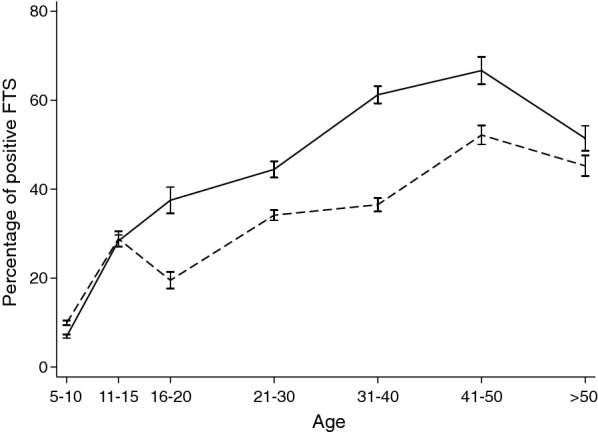
Age-profiles for *W. bancrofti* antigenaemia prevalence. Males (solid line), females (dashed line). Bars indicate 95% confidence intervals

The proportion of subjects with CFA in the study population was 31.6% (259/820). The prevalence was slightly higher in males than in females (34.2 *vs* 29.3%, respectively; Pearson’s Chi-square test: *χ*^2^ = 2.28, *df* = 1, *P* = 0.131) and increased linearly with age for both sexes leveling off at 40–50 years of age (Fig. 2).

### Microfilaraemia

**Fig. 3:**
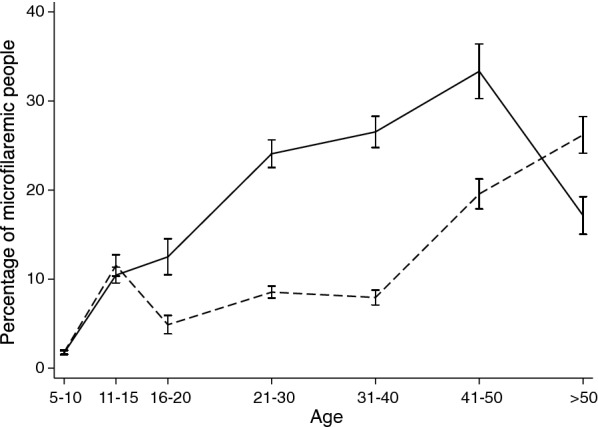
Age-profiles for *W. bancrofti* microfilarial prevalence. Males (solid line), females (dashed line). Bars indicate 95% confidence intervals

Microfilaria were detected in night blood from 37% (97/259) of participants with positive FTS results. The *W. bancrofti* mf prevalence was 11.8% (97/820), with a higher value in males than in females (14.4 *vs* 9.6%; Pearson’s Chi-square test: *χ*^2^ = 4.41, *df* = 1, *P* = 0.036). Microfilaraemia prevalence increased linearly with age between 5 and 50 years in males, while a sharp increase in mf prevalence was observed in females after age 40 (Fig. 3).

### Risk factors for filarial antigenaemia

**Fig. 4:**
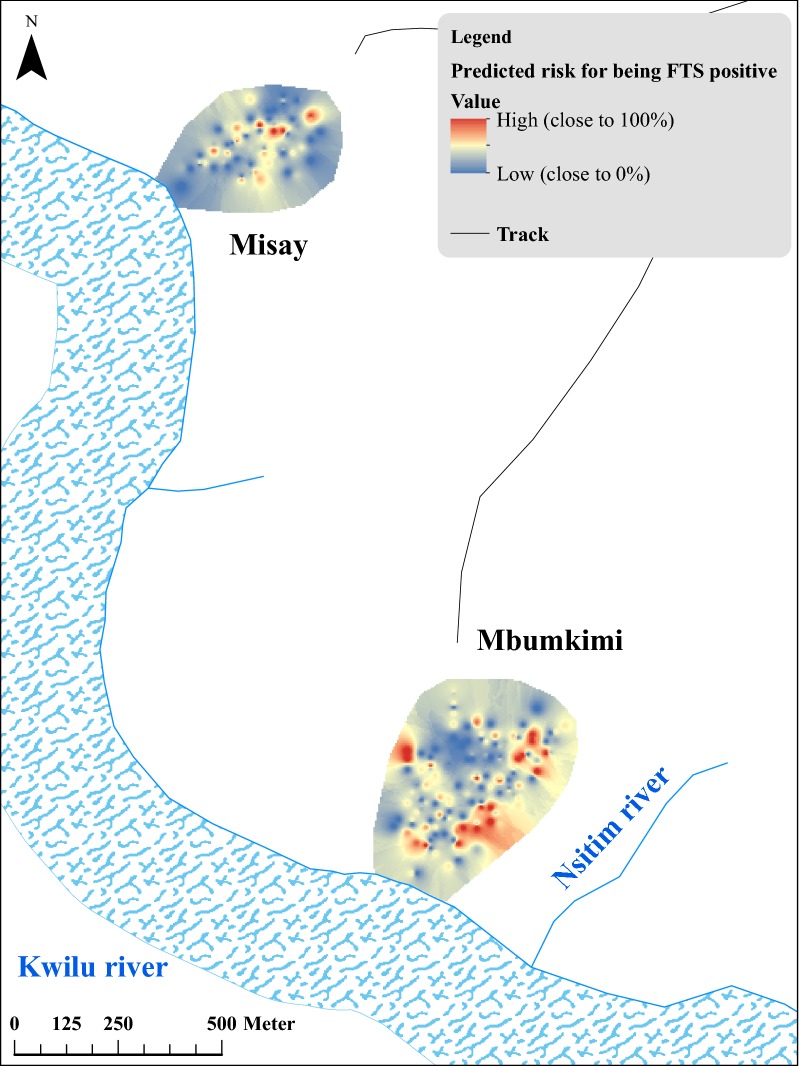
Interpolation by kernel density for the total population

In the final model (Table 3), the risk of filarial antigenaemia increased with age (adjusted odd ratio aOR = 9.12, 95% confidence interval CI: 4.47–18.61, *P* < 0.001, in those aged > 35 years, when compared with individuals aged 5–10 years, *P* < 0.001). Males had a higher CFA prevalence than females (aOR = 1.75, 95% CI: 1.20–2.53, *P* = 0.003). People who (i) did not use bednets, (ii) performed agricultural activities, (iii) had not taken anthelmintics during the past year, and (iv) lived near the Nsitim River (Fig. 4, kernel interpolation), had a significantly increased risk for CFA (Table 3). Random household effect, representing all unmeasured contextual effects common within a household (e.g. habits, genetic component), was significant (likelihood-ratio Chi-bar-square test: *χ*^2^ = 6.78, *df* = 13, *P* = 0.005). Lastly, the calculation of the attributable parts of each variable (Table 4) indicates that agricultural activities were responsible for the highest PAF value (30.5%, 95% CI: 7.4–47.9), and that the total PAF related to activities outside of the villages (e.g. agricultural activities plus staying overnight in the bush) was 36.0%. Similarly, the total PAF due to factors internal to the village (use of bednets plus proximity to the river) was 18.9%. Interestingly, not having taken anthelmintics was found to have an important impact (PAF = 25.7%, 95% CI: 8.9–39.4). When models were run to consider the different drugs taken by the subject, only history of levamisole treatment was significantly associated with a decreased risk for CFA (aOR = 0.40, 95% CI: 0.21–0.76, *P* = 0.005) (Table 5). Among those taking levamisole, no drugs, mebendazole, and albendazole, 16.2, 34.8, 30.2, and 30.8% were FTS positive, respectively (Pearson’s Chi-square test: *χ*^2^ = 15.64, *df* = 3, *P* = 0.002). Interestingly, a history of levamisole treatment was more common in the youngest age group (Pearson’s Chi-square test: *χ*^2^ = 50.84, *df* = 9, *P* < 0.0001). There was no interaction between the drug variable and age in an analysis that used anthelmintic drugs as a binary variable (likelihood-ratio Chi-square test: *χ*^2^ = 5.71, *df* = 3, *P* = 0.769) or as a categorical variable by name of drug (likelihood-ratio Chi-square test: *χ*^2^ = 1.24, *df* = 9, *P* = 0.743).

**Table 3:** Multivariable logistic regression analysis of risk factors for filarial antigenaemia in the total population. Likelihood-ratio test for random effect *vs* fixed effect model, final model (*P* = 0.005)

Variable	Category	Full model		Final model	
		Adj OR (95% CI)	*P*	Adj OR (95% CI)	*P*
Age (ref: 5–10 years-old)	11–20	2.72 (1.42–5.20)	0.002	3.03 (1.59–5.75)	0.001
	21–35	5.19 (2.53–10.65)	<0.001	5.46 (2.68–11.12)	0.001
	> 35	8.23 (4.05–16.73)	<0.001	9.12 (4.47–18.61)	<0.001
Sex (ref: female)	Male	1.82 (1.25–2.66)	0.002	1.75 (1.20–2.53)	0.003
Village (ref: Mbumkimi)	Misay	0.92 (0.54–1.56)	0.758		
Bednets (ref: yes)	No	1.46 (0.99–2.14)	0.056	1.57 (1.06–2.33)	0.023
Latrines (ref: no)	Yes	0.88 (0.54–1.43)	0.609		
Fishing (ref: no)	Yes	1.27 (0.83–1.94)	0.277		
Occasional stay in the bush (ref: no)	Yes	1.35 (0.86–2.11)	0.186	1.45 (0.92–2.28)	0.105
Agriculture activity (ref: no)	Yes, same side	2.10 (1.07–4.11)	0.031	2.15 (1.11–4.17)	0.024
	Yes, other side	2.07 (1.15–3.71)	0.015	2.21 (1.25–3.90)	0.006
Previous AH treatment (ref: yes)	No	1.77 (1.13–2.77)	0.012	1.82 (1.16–2.87)	0.010
	na	2.10 (0.89–4.96)	0.090	2.05 (0.84–4.94)	0.111
Population density (ref: low)	Intermediate	0.99 (0.57–1.71)	0.969		
	High	1.31 (0.77–2.25)	0.322		
	na	1.51 (0.59–3.87)	0.387		
Distance to Nsitim River (ref: > 400 m)	300–400 m	1.40 (0.84–2.34)	0.198	1.41 (0.89–2.21)	0.140
	0–300 m	2.44 (0.99–6.01)	0.052	2.78 (1.14–6.74)	0.024
Distance to Kwilu River (ref: > 400 m)	300–400 m	0.64 (0.32–1.30)	0.216		
	0–300 m	1.23 (0.64–2.36)	0.543		
Number of household members		1.04 (0.93–1.18)	0.475		

*Abbreviations*: Adj OR, adjusted odds ratio; AH, anthelmintic drug; CI, confidence interval; na, not available

**Table 4:** Population attributable fractions associated with the significant independent variables for filarial antigenaemia and microfilaraemia

Variable	Antigenaemia			Microfilaraemia		
	Population attributable fraction	95% CI	*P*	Population attributable fraction	95% CI	*P*
Males	12.7	3.7–20.9	0.006	21.7	3.6–36.4	0.021
No bednet	9.4	1.8–16.3	0.016	–	–	–
No previous AH treatment	25.7	8.9–39.4	0.004	43.3	12.0–63.5	0.011
Occasional stay in the bush	5.5	0.1–11.3	0.078	17.0	2.8–29.1	0.020
Agricultural activity	30.5	7.4–47.9	0.013	–	–	–
Distance to Nsitim < 400 m	9.4	0.8–17.3	0.034	20.7	2.7–35.3	0.027

*Abbreviation*: AH, anthelmintic drug; CI, confidence interval

**Table 5:** Multivariable logistic regression analysis of risk factors for filarial antigenaemia and microfilaraemia in the total population

Variable	Category	Antigenaemia model^a^				Microfilaraemia model^a^			
		Full model		Final model		Full model		Final model	
		Adj OR (95% CI)^b^	*P*	Adj OR (95% CI)^c^	*P*	Adj OR (95% CI)^b^	*P*	Adj OR (95% CI)^d^	*P*
Previous AH treatment	Levamisole (*n* = 117)	0.41 (0.22–0.76)	0.005	0.40 (0.21–0.76)	0.005	0.40 (0.16–0.98)	0.046	0.41 (0.17–0.99)	0.048
(ref: no)	Mebendazole (*n* = 53)	0.75 (0.35–1.61)	0.460	0.77 (0.35–1.69)	0.509	0.52 (0.17–1.52)	0.237	0.56 (0.19–1.64)	0.291
	Albendazole (*n* = 52)	0.79 (0.37–1.65)	0.525	0.73 (0.34–1.55)	0.414	0.36 (0.10–1.29)	0.115	0.35 (0.10–1.24)	0.104
LR test random effect			0.065		0.005		0.252		0.197

^a^As only one individual had taken pyrantel, the models were performed on 819 individuals (the subject having taken pyrantel was considered as missing data)

^b^Adjusted on age, sex, latrine, bednets, village, occasional overnight stay in the bush, agricultural activity, fishing, distance to the Nsitim and Kwilu rivers, population density and number of household members

^c^Adjusted on age, sex, occasional overnight stay in the bush, bednets, distance to the Nsitim and Kwilu rivers, and agricultural activity

^d^Adjusted on age, sex, occasional overnight stay in the bush, and distance to the Nsitim River

*Abbreviations*: Adj OR, adjusted odds ratio; AH, anthelmintic drug; CI, confidence interval; LR, likelihood ratio

### Risk factors for *W. bancrofti* microfilaraemia

In the final model, age was a strong risk factor for microfilaraemia (aOR = 12.68, 95% CI: 4.22–38.07, for persons > 35 years of age) (Table 6). Male sex was linked to a higher prevalence of mf (aOR = 1.86, 95% CI: 1.15–3.01, *P* = 0.011). Persons who occasionally stayed overnight in the bush (aOR = 2.01, 95% CI: 1.18–3.42, *P* = 0.010), who had not taken anthelmintics (aOR = 2.50, 95% CI: 1.33–4.72, *P* = 0.005), and who lived near the Nsitim River (aOR = 2.73, 95% CI: 1.04–7.11, *P* = 0.046) had an increased risk for mf. Household effect was not significant (Likelihood-ratio Chi-bar-square test: *χ*^2^ = 0.41, *df* = 10, *P* = 0.260). Lastly, the PAF related to previous anthelmintic treatment suggested that if all people in the study had taken levamisole within the past year, the mf prevalence in the study villages might have been 43.3% lower than what was observed (Table 4). As seen in the CFA analyses, only levamisole was significantly associated with a decreased risk for mf (aOR = 0.41, 95% CI: 0.17–0.99, *P* = 0.048) (Table 5). However, when analyses were restricted to the 259 FTS positive individuals, only overnight stays in the bush tended to be associated with mf (aOR = 1.69, 95% CI: 0.94–3.03, *P* = 0.078). Consequently, levamisole (or other anthelmintic treatment) was not significantly associated with mf in individuals infected with *W. bancrofti*.

**Table 6:** Multivariable logistic regression analysis of risk factors for microfilaraemia in the total population. Likelihood-ratio test for random effect *vs* fixed effect model, final model (*P* = 0.260)

Variable	Category	Full model		Final model	
		Adj OR (95% CI)	*P*	Adj OR (95% CI)	*P*
Age (ref: 5–10 years-old)	11–20	4.07 (1.26–13.03)	0.018	5.02 (1.64–15.32)	0.005
	21–35	5.87 (1.73–20.00)	0.005	7.78 (2.57–23.56)	0.001
	> 35	9.37 (2.84–30.92)	0.001	12.68 (4.22–38.07)	0.001
Sex (ref: female)	Male	1.99 (1.19–3.33)	0.008	1.86 (1.15–3.01)	0.011
Village (ref: Mbumkimi)	Misay	1.25 (0.60–2.58)	0.554		
Bednets (ref: yes)	No	0.94 (0.57–1.56)	0.816		
Latrines (ref: no)	Yes	1.05 (0.54–2.07)	0.882		
Fishing (ref: no)	Yes	1.00 (0.56–1.76)	0.993		
Occasional stay in the bush (ref: no)	Yes	1.92 (1.11–3.35)	0.020	2.01 (1.18–3.42)	0.010
Agricultural practice (ref: no)	Yes, same side	1.65 (0.62–4.44)	0.312		
	Yes, other side	1.67 (0.72–3.87)	0.235		
Previous AH treatment (ref: yes)	No	2.54 (1.33–4.84)	0.005	2.50 (1.33–4.72)	0.005
	na	0.94 (0.19–4.63)	0.939	0.78 (0.16–3.82)	0.757
Population density (ref: low)	Intermediate	1.32 (0.65–2.69)	0.446		
	High	1.65 (0.82–3.34)	0.163		
	na	1.77 (0.48–6.55)	0.389		
Distance to Nsitim River (ref: > 400 m)	300–400 m	2.37 (1.20–4.68)	0.013	1.97 (1.17–3.33)	0.011
	0–300 m	3.02 (1.02–8.89)	0.046	2.73 (1.04–7.11)	0.041
Distance to Kwilu River (ref: > 400 m)	300–400 m	1.00 (0.40–2.54)	0.989		
	0–300 m	1.52 (0.65–3.54)	0.335		
Number of household members		0.99 (0.85–1.15)	0.908		

*Abbreviations*: Adj OR, adjusted odds ratio; AH, anthelmintic drug; CI, confidence interval; na, not available

## Discussion

Little is known regarding the risk factors associated with LF in Central Africa and its micro-epidemiology. Results from this study confirm some findings from a similar study that was conducted in the village of Seke Pembe, in the Republic of Congo [5], namely that the risk of infection with *W. bancrofti* increased with age, and males had higher infection prevalences than females, independent of the other variables. These profiles are commonly seen in other areas around the world. The use of bednets was a protective factor against LF infection, and anthelmintic treatment during the past year appeared to be strongly protective, especially regarding mf prevalence. There was no difference in bednet use for individuals who had or had not taken anthelmintics (Pearson’s Chi-square test: *χ*^2^ = 0.0016, *df* = 1, *P* = 0.968), and there was no interaction between these two variables (likelihood-ratio Chi-square test: *χ*^2^ = 0.48, *df* = 1, *P* = 0.489); this suggests that bednets and anthelmintic treatment had independent protective effects.

The protective effect of anthelmintics appeared to be only significant for levamisole. Published studies of the effects of levamisole for *W. bancrofti* and *Brugia malayi* infections show that it temporarily reduces mf densities (ranging from 78.8 to 98.5%) [12–16]. However, while levamisole is known to have a transient microfilaricidal effect, none of these trials suggested that it has a macrofilaricidal effect. Thus, the lower CFA prevalence in persons who reported levamisole use was unexpected. It is possible that levamisole has previously unrecognized macrofilaricidal or prophylactic effects on *W. bancrofti.* This unexpected result should be investigated more thoroughly, because we cannot exclude possible confounding factors at this time, and because this study was not specifically designed to assess the effect of anthelminthic drugs on the acquisition of filarial infection. We cannot exclude a possible similar protective effect of albendazole since relatively few people reported having taken albendazole during the year prior to our survey.

Our study identified several activities as risk factors for CFA and microfilaraemia. Infection was more frequent in individuals who worked in agriculture. However, the risk of infection was not higher in persons who worked in fields located across the Kwilu River. This suggests that frequent river crossings did not increase workers’ exposure to infective mosquitoes. This conclusion is consistent with the finding that proximity of households to the Kwilu River was not significantly associated with a higher risk of infection. The PAF of risk for infection associated with agricultural activity was second only to anthelmintic intake, the latter of which might be considered to be an intervention rather than a risk factor. In addition, given the link between agricultural activity and LF test positivity, it would be of interest to try to elucidate the causal mechanism driving this relationship. These could identify possibly beneficial interventions for the population.

It is interesting to note that results from this study, which suggest a large amount of LF transmission occurs outside of the village, are consistent with results of our prior study in Republic of Congo [5]. A history of frequent fishing was not associated with the presence of infection, with or without adjusting for age, sex or agricultural activity. This result may be because most fishing activities occur during the day and not in the evening when exposure to *Anopheles* mosquitoes is highest.

The population density around houses and the number of household members were not associated with infection risk. This result suggests that there was no dilution or concentration effect on transmission related to the domestic or peridomestic environment. This finding also suggests that transmission does not mainly occur in or near residences. Studies in east Africa reported that *Anopheles* densities decrease significantly beyond 400 m from water sites [17, 18]. Similarly, a meta-analysis showed that increasing distance away from breeding sites was associated with an 11% reduction in malaria risk per 100 m [19]. We have limited information on the mosquito fauna in the study area. Studies in and around Bandundu-ville detected abundant *A. gambiae* (*s.l.*) [20], especially complex member *A. coluzzii* (*s.s.*) [21]. However, *A. funestus* (*s.l.*), and to a lesser extent, *A. moucheti* (*s.l.*) and *A. nili* (*s.l.*) might also be significant vectors for LF in the study area [22].

It is interesting that proximity to the Nsitim River was a risk factor while proximity to the Kwilu River was not. The Kwilu is a large river (200 m wide), while the Nsitim River is a small stream that flows slowly in a dense gallery forest. The local population goes to this small river for bathing, for washing clothes and for collecting drinking water. The access to the Nsitim is swampy and no crops are grown in this area. Thus, this environment may be favorable for specific *Anopheles* species such as *A. moucheti* (*s.l.*) or *A. nili* (*s.l*.) that breed in slow-flowing rivers [23, 24]. Further entomological studies are needed to identify LF vectors in Central Africa.

It was also interesting that staying overnight in the bush was a risk factor for mf. It is likely that this activity increases exposure to mosquitoes. This risk factor was also identified in our prior study in the Republic of Congo [5]. People who acquire filarial infections in the bush may constitute an important reservoir for secondary transmission inside the village. These results from two studies in different countries suggest that sylvatic or peri-sylvatic exposure to mosquitoes is important for LF transmission in Central Africa [5]. More work is needed to compare vector species and densities in villages and forested bush areas in LF endemic areas in this region.

## Conclusions

This study has provided useful information on the epidemiology of LF in Central Africa. Age, sex, environment, and individual behavior were important risk factors for infection, and these findings were consistent with those recently reported from a study in the Republic of Congo. Additional studies with strong entomology components should be conducted to further define the epidemiology and explain the highly focal distribution of LF in this important region.

## Abbreviations

GPELF: Global Programme to Eliminate Lymphatic Filariasis
MDA: mass drug administration
LF: lymphatic filariasis
IVM: ivermectin
ALB: albendazole
NTD: neglected tropical disease
FTS: filariasis test strip
CFA: circulating filarial antigens
mf: microfilariae
DOLF: Death to Onchocerciasis and Lymphatic Filariasis
